# Supplementary material for: The Respiratory Burst Oxidase Homolog Protein D (GhRbohD) Positively Regulates the Cotton Resistance to Verticillium dahliae
Source: Int J Mol Sci. 2021 Dec 2;22(23):13041. doi: 10.3390/ijms222313041 (PMC8657740; doi:10.3390/ijms222313041)
Supplement: Supplementary file 1 [file ijms-22-13041-s001.zip › ijms-1494531-supplementary.pdf]

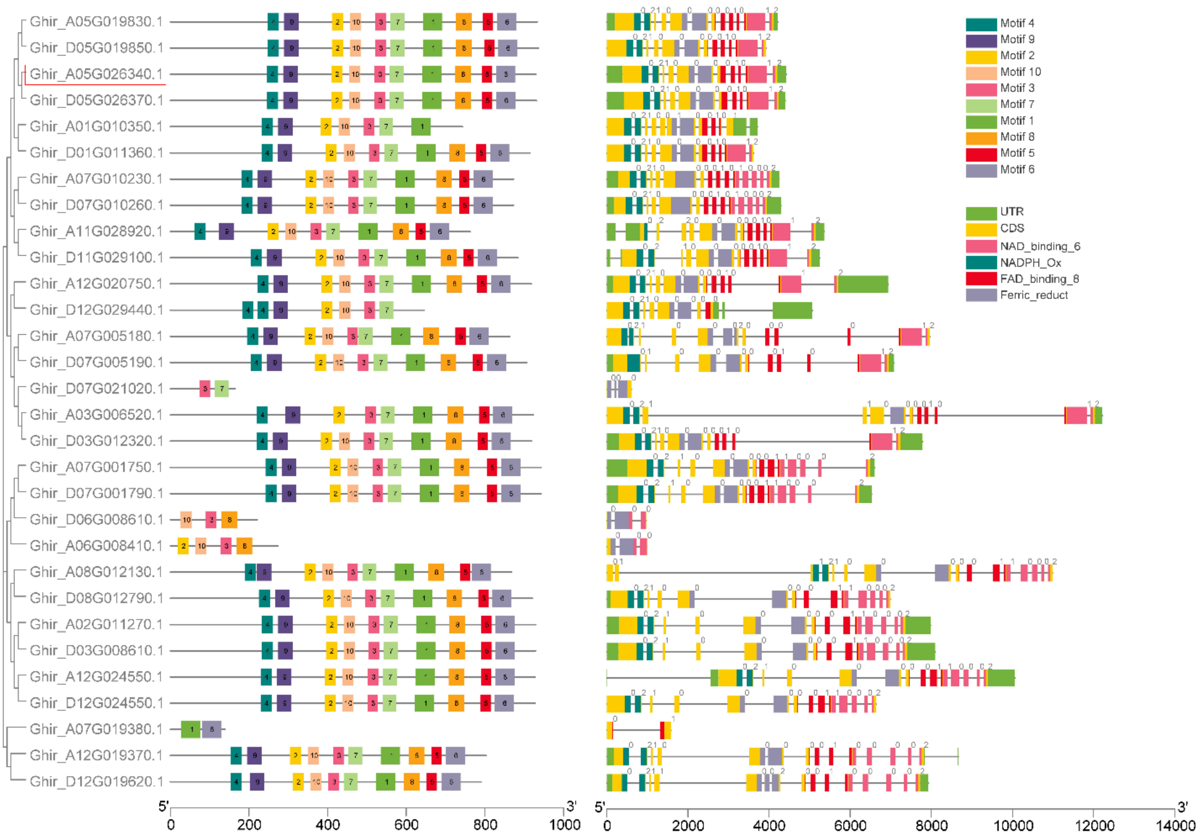

Supplementary Figure S1. Gene structure and conserved domain analysis of Rbohs in *Gossypium hirsutum*. The Rboh proteins conserved domain architecture. Motifs were identified by a MEME analysis (http://meme-suite.org/) and proteins length was estimated via the scale.

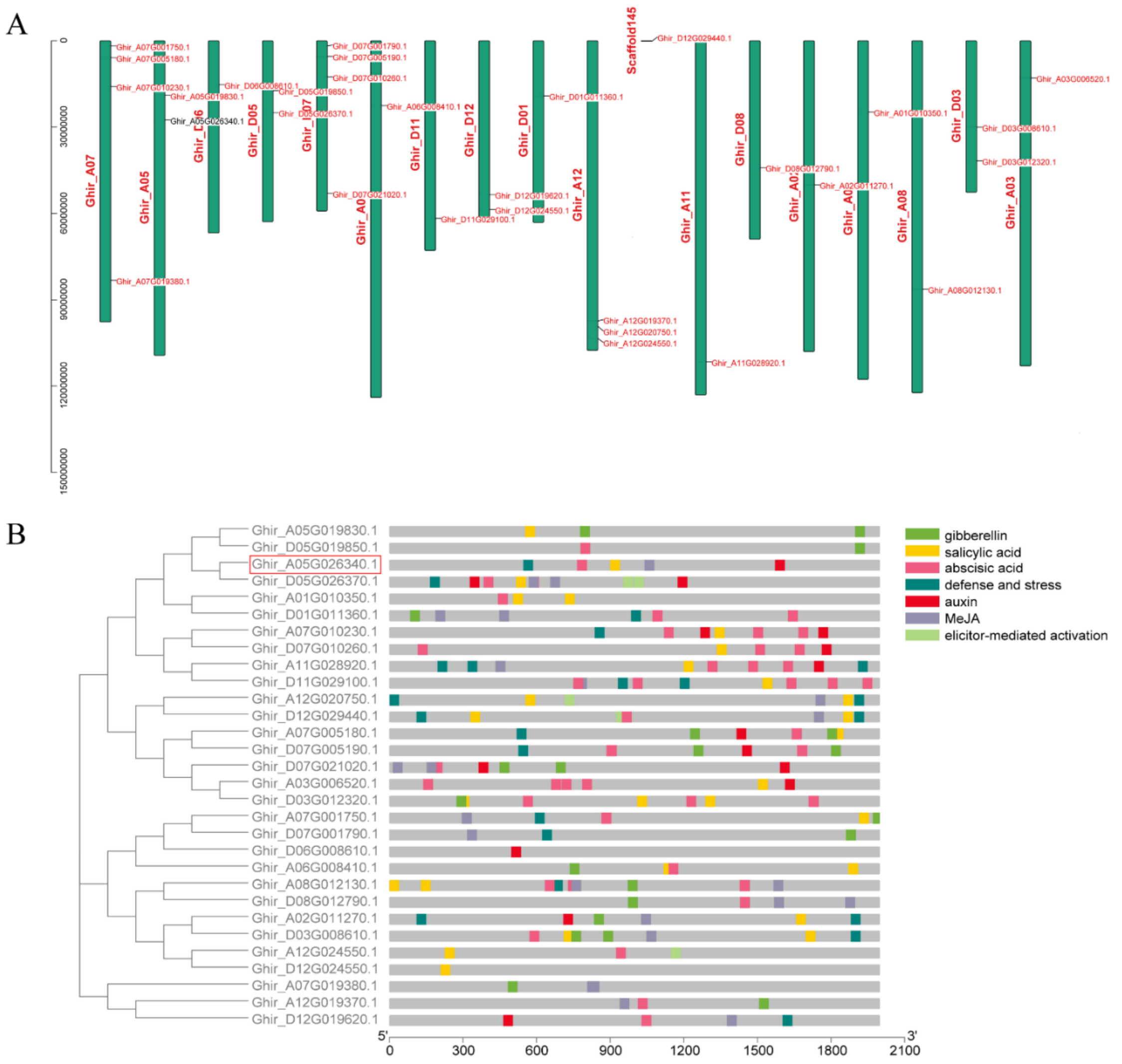

Supplementary Figure S2. (A) TBtools software was used for gene chromosomal localization analysis, and GhRbohD (Ghir_A05G263401) was located on chromosome A05. (B) The promoter sequence of GhRbohD family genes was extracted and cis-acting elements were analyzed to obtain cis-acting elements related to disease resistance.

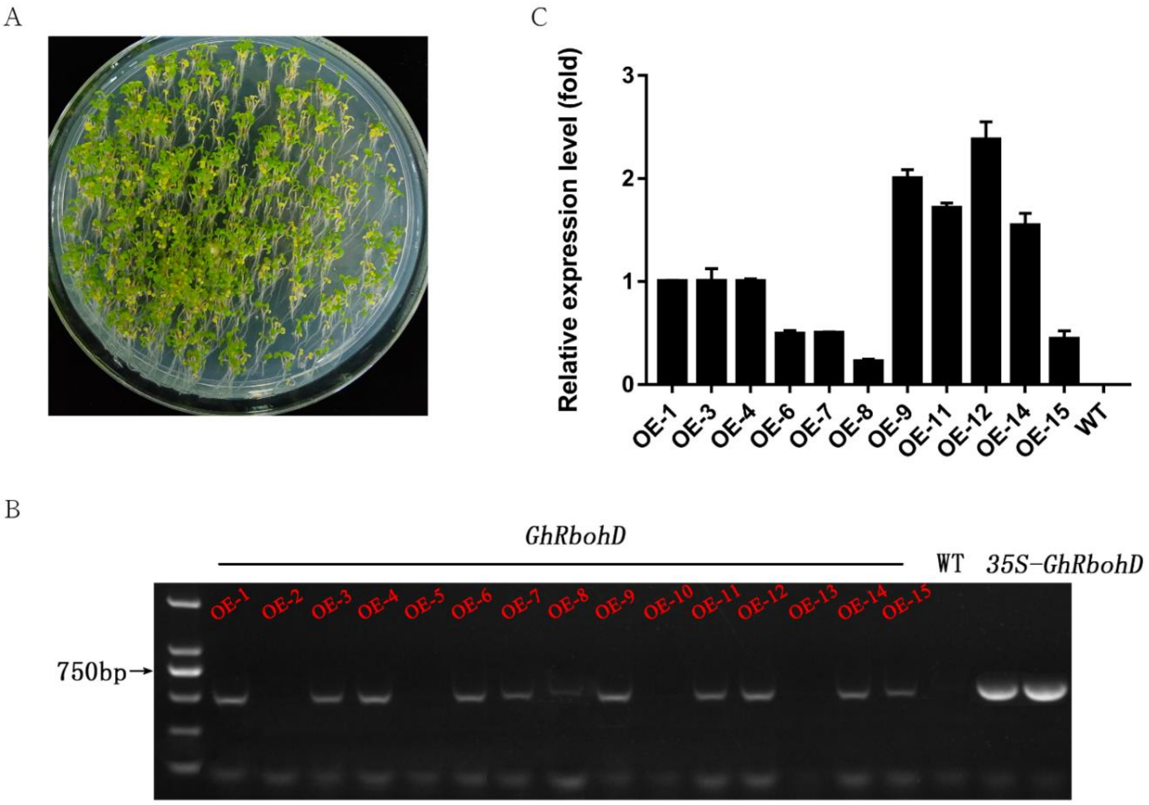

Supplementary Figure S3. PCR detection of transgenic *Arabidopsis thaliana*. 35S-GhRbohD is the plasmid, as a positive control; WT is wild-type *Arabidopsis thaliana* as a negative control; The GhRbohD with the corresponding band is the selected overexpression plant.

Supplementary table 1. Primers used in this research

Primer name	Sequences(5'-3')	Destination
GhRbohD-full-F	ATGAGGAACGATGATGGGAGAG	Gene cloning
GhRbohD-full-R	TTAAAAATTTTCTTTGTGGAATTCG	
GhRbohD-VIGS-F	CGGAATTCTACTGGGTGACAAGGGAGCA	VIGS
GhRbohD-VIGS-R	GCTCGAGTAGGCTTGGCGAAGTGAGAT	
GhRbohD-OE-F	CGGGATCCATGAGGAACGATGATGGGAGAG	overexpression
GhRbohD-OE-R	TCCCCCCGGGAAAATTTTCTTTGTGGAATTCGAAC	
GhCHI-F	AATGACACGGCGACTCCCTT	qRT-PCR
GhCHI-R	TCCCACGAACCCCCACCTAT	
GhJaz1-F	AGCCTCAAAAAGGAAGACCTCAAAC	qRT-PCR
GhJaz1-R	TGGCTGCTCAATCACCATAGTAATC	
GhNOA1-F	GAGGATGCTGAAAGACCTGCTA	qRT-PCR
GhNOA1-R	TCTCAACTGGCTTGGGTACATG	
GhC4H1-F	CCGAACCCGACACCCATAAGC	qRT-PCR
GhC4H1-R	GCAGGGATGTCATACCCACCAAG	
GhPAL5-F	CCAAGTGGCTAAAAGAGTCCTAACA	
GhPAL5-R	GGTCGTCGGCGTAGGCATAGA	
GhPR3-F	GATGACTCCACAATCACCGAAGC	
GhPR3-R	GCGGTCTTCTACCTGGGCATT	
GhHIN1-F	GCTGATGAGACATCGGAGTTTA	
GhHIN1-R	CTACCATTCCCAGTGTTCAAAG	
GhRbohD-35S-F	CGACAGTGGTCCCAAAGATG	PCR of transgenic *Arabidopsis thaliana*
GhRbohD-35S-R	CGTAGAGGCGGTCAAGTCAT	
GhRbohD-BD-F	CATGGAGGCCGAATTCATGAGGAACGATGATGGG	pGBKT7-RbohD for
GhRbohD-BD-R	GCAGGTCGACGGATCCTTAAAAATTTTCTTTGTGGAATTCG	Y2H
GhPBL9-AD-F	GGAGGCCAGTGAATTCATGGGGTCTTGTTTTAGTTCTAGAA	pGADT7-PBL9 for
GhPBL9-AD-R	TTCATCTGCAGCTCGAGCTCTTATGTACTTAATGGAGATGCAGA	Y2H
GhRPL12C-AD-F	GGAGGCCAGTGAATTCATGCCGCCGAAGTTTGACC	pGADT7-RPL12C
GhRPL12C-AD-R	TTCATCTGCAGCTCGAGCTCTCACTCCAAGGGAACATCGAC	for Y2H
